# The Role of Alternative Oxidase in the Interplay between Nitric Oxide, Reactive Oxygen Species, and Ethylene in Tobacco (*Nicotiana tabacum* L.) Plants Incubated under Normoxic and Hypoxic Conditions

**DOI:** 10.3390/ijms23137153

**Published:** 2022-06-28

**Authors:** Somaieh Zafari, Greg C. Vanlerberghe, Abir U. Igamberdiev

**Affiliations:** 1 Department of Biology, Memorial University of Newfoundland, St. John’s, NL A1C 5S7, Canada; 2 Department of Biological Sciences, University of Toronto Scarborough, Toronto, ON M1C 1A4, Canada; greg.vanlerberghe@utoronto.ca; 3 Department of Cell and Systems Biology, University of Toronto Scarborough, Toronto, ON M1C 1A4, Canada

**Keywords:** *Nicotiana tabacum* L., alternative oxidase, ethylene, hypoxia, nitric oxide, reactive oxygen species

## Abstract

The transgenic tobacco (*Nicotiana tabacum* L.) plants with the modified levels of alternative oxidase (AOX) were used to evaluate the physiological roles of AOX in regulating nitro-oxidative stress and metabolic changes after exposing plants to hypoxia for 6 h. Under normoxia, AOX expression resulted in the decrease of nitric oxide (NO) levels and of the rate of protein *S*-nitrosylation, while under hypoxia, AOX overexpressors exhibited higher NO and *S*-nitrosylation levels than knockdowns. AOX expression was essential in avoiding hypoxia-induced superoxide and H_2_O_2_ levels, and this was achieved via higher activities of catalase and glutathione reductase and the reduced expression of respiratory burst oxidase homolog (Rboh) in overexpressors as compared to knockdowns. The AOX overexpressing lines accumulated less pyruvate and exhibited the increased transcript and activity levels of pyruvate decarboxylase and alcohol dehydrogenase under hypoxia. This suggests that AOX contributes to the energy state of hypoxic tissues by stimulating the increase of pyruvate flow into fermentation pathways. Ethylene biosynthesis genes encoding 1-aminocyclopropane-1-carboxylic acid (ACC) synthase, ACC oxidase, and ethylene-responsive factors (ERFs) were induced during hypoxia and correlated with AOX and NO levels. We conclude that AOX controls the interaction of NO, reactive oxygen species, and ethylene, triggering a coordinated downstream defensive response against hypoxia.

## 1. Introduction

Nitric oxide (NO) appears to be a critical signaling and metabolic molecule in a wide range of environmental crises, such as hypoxia. When the ambient oxygen (O_2_) is low, NO production and its turnover are involved in the phytoglobin/nitric oxide pathway (Pgb/NO cycle) for the maintenance of redox and energy levels [1,2]. NO represents the main reactive nitrogen species (RNS), and it can interact with reactive oxygen species (ROS) such as superoxide anion (O_2_^−^), forming other reactive nitrogen species, e.g., peroxynitrite (ONOO^−^). The main sources of ROS in cells are the plasma membrane NADPH oxidases, or respiratory burst oxidase homologs (Rbohs) [3]. They catalyze the apoplast generation of O_2_^−^, which is then converted into hydrogen peroxide (H_2_O_2_). Even though NO and ROS are produced under normal physiological conditions, they are prone to uncontrolled overproduction in stressful situations, resulting in cellular nitro-oxidative damage that can compromise cell survival.

Plant cells have evolved sophisticated defense mechanisms to avoid organelle damage as a result of redundant NO and ROS formation, e.g., excessive electron flow is dissipated by the alternate respiratory pathways, such as AOX, to preserve mitochondrial redox state. AOX maintains energy stability and keeps the flow through the mitochondrial electron transport chain (mETC) by minimizing the generation of mitochondrial NO and ROS [4,5]. However, under hypoxic conditions, AOX activity accelerates NO generation in tobacco, followed by NO conversion to nitrate by the upregulated class 1 phytoglobin (Pgb1) [2]. AOX may also play a crucial role in ROS avoidance via activating antioxidant enzymes superoxide dismutase (SOD), catalase (CAT), ascorbate peroxidase (APX), and guaiacol peroxidase (GPX), as revealed in brassinosteroids-treated *Cucumis sativus* [6]. Despite the current knowledge regarding the AOX contribution to hypoxia, the NO and ROS interplay in plants expressing variable levels of AOX under hypoxic conditions is little understood.

Plants have evolved a plethora of plant-specific transcription factors to precisely control the expression of their genes. The ethylene response factors (ERFs) superfamily is one of the largest families of transcription factors that respond to a variety of biotic and abiotic stressors by binding directly to promoter regions of defense-related genes [7]. Overexpression of hypoxia-inducible ERF genes promotes tolerance to low-oxygen environments in *Arabidopsis* and rice [8,9]. Since many ERFs are involved in stress responses, the tight control of ERFs ensures that plants respond to environmental stressors effectively. Ubiquitin-mediated protein degradation regulates ERF proteins stability via the 26S proteasome pathway. The N-end rule route is also responsible for the stability of ERFs belonging to the group VII ethylene response factors (ERF-VII) [10]. ERF-VII proteolysis is facilitated by oxygen and NO [11], while ethylene (ET) promotes their stability [12]. ET was found to be critical for the improved hypoxia tolerance by increasing the expression of genes encoding proteins involved in fermentation, energy maintenance, oxidative stress, NO scavenging, and O_2_ sensing [11,13]. Studies have provided solid evidence that ET-signaling pathways are essential for the stress-induced upregulation of AOX [14,15]. In this work, the effects of different amounts of AOX on the expression of ERFs and ET biosynthesis enzymes (1-aminocyclopropane-1-carboxylic (ACC) synthase (ACS) and ACC oxidase (ACO)) were investigated in transgenic tobacco under hypoxia. Furthermore, the molecular mechanism by which AOX impacts the interaction of NO, ROS, and ET in alleviating hypoxic-induced stress in tobacco was also examined.

## 2. Results

### 2.1. NO Production and Protein S-Nitrosylation under Hypoxia

**Figure 1:**
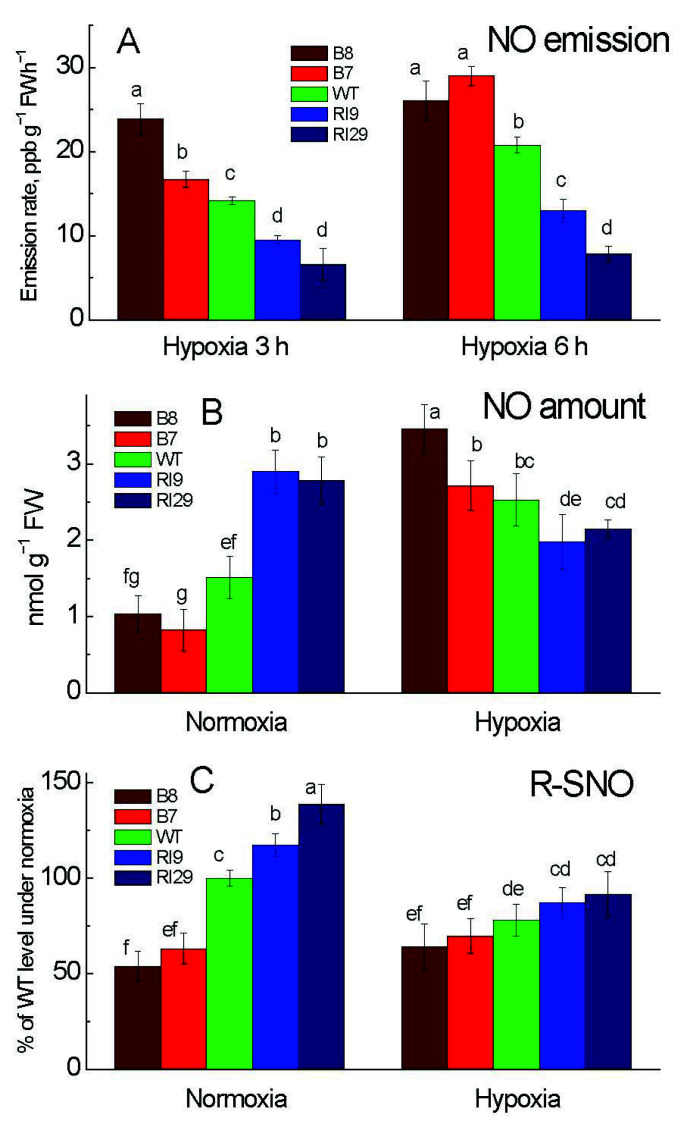
Leaf nitric oxide emission (**A**), total NO amount (**B**), and *S*-nitrosylation level (**C**) in tobacco plants with differing amounts of alternative oxidase and exposed to normoxia or hypoxia (6 h, unless indicated different). The plant lines used included wild type (WT), two alternative oxidase overexpressors (B7 and B8), and two alternative oxidase knockdowns (RI9 and RI29). Vertical bars indicate SD from three to four independent experiments (*n* = 3–4); different letters indicate significant differences between five tobacco lines and the treatments.

Tobacco leaves, when placed under hypoxia, emitted NO, while NO emission from leaves incubated in air could not be detected by using the method of chemiluminescence, due to technical features of this method [16]. From three to six hours of hypoxia, we observed an increase in NO emission in all plant lines. AOX overexpressors (B7 and B8) had much greater NO rates than the WT, which exhibited an intermediate rate, while the NO emission rates of the AOX knockdown lines (RI9 and RI29) were significantly lower than those of the WT (Figure 1A).

To confirm these results by the second method [16] and to determine the amount of NO in the leaves exposed to normal air, we used an independent biochemical method to measure leaf NO content, which includes the interaction of NO with hemoglobin. Under normoxia, NO concentrations in RI29 and RI9 were nearly two times greater than in WT. The NO amount in the B7 was lower than that in the WT (and B8), while the mean NO amount in B8 was only slightly lower (and not significantly different) than that in WT (Figure 1B). The level of NO in the overexpressors and WT increased significantly in hypoxic conditions but dropped in the knockdowns. Now, when compared to WT, NO levels were higher in AOX overexpressors (particularly B8) and lower in AOX knockdowns (particularly RI29), as is consistent with the chemiluminescence results (Figure 1A).

Under normoxia, the level of protein *S*-nitrosylation (R-SNO) was significantly lower in the AOX overexpressors, as compared to the WT and knockdowns (Figure 1C). The two knockdowns had consistently higher concentrations of R-SNO than the WT. In this case, the concentration of R-SNO was also considerably higher in the stronger knockdown (RI29) than the slightly leaky AOX knockdown (RI9). In response to hypoxia, the R-SNO amount decreased in the WT and knockdown plants but did not change significantly in overexpressors. When compared to WT, the level of R-SNO in the overexpressors was only slightly lower and somewhat higher in the knockdowns (but not significantly different).

### 2.2. ROS Production and Cell Damage under Hypoxia 

**Figure 2:**
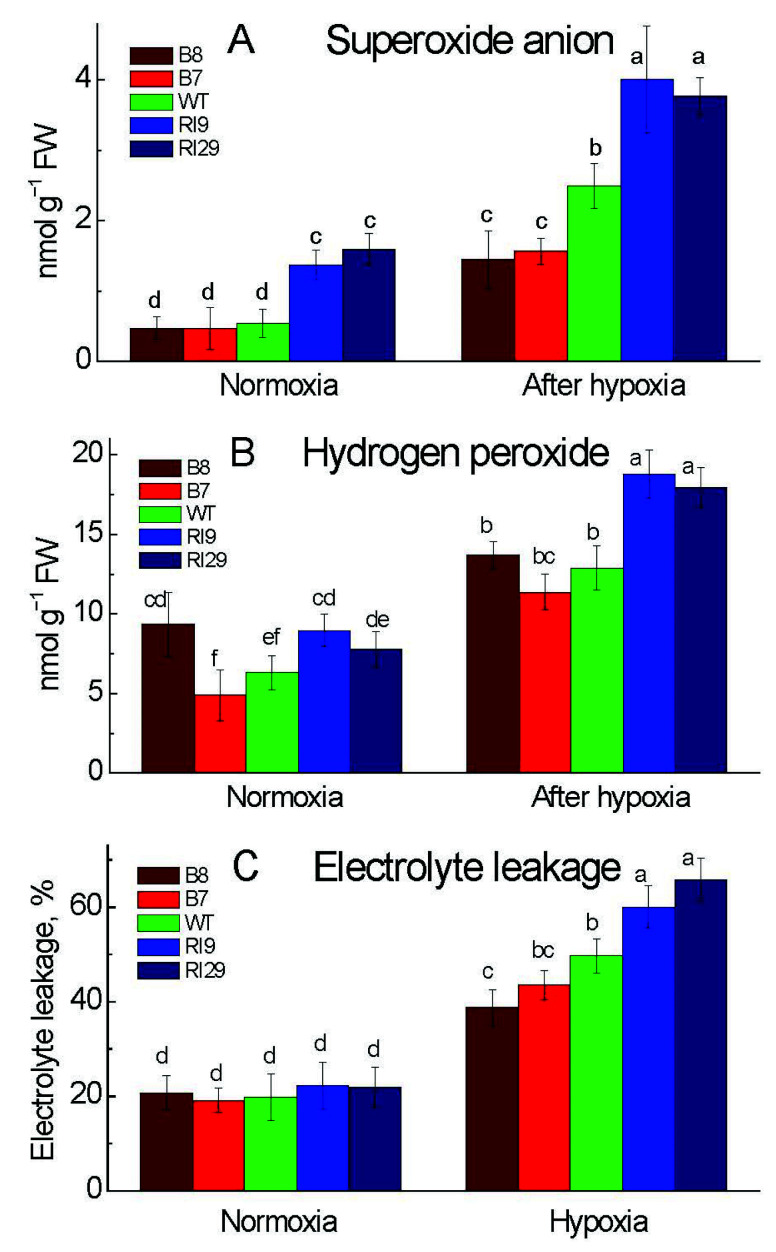
Leaf superoxide anion level (**A**), hydrogen peroxide amount (**B**), and electrolyte leakage rate (**C**) in tobacco plants with differing amounts of alternative oxidase and exposed to normoxia or 6 h hypoxia. The plant lines used included wild type (WT), two alternative oxidase overexpressors (B7 and B8), and two alternative oxidase knockdowns (RI9 and RI29). Vertical bars indicate SD from three to four independent experiments (*n* = 3–4); different letters indicate significant differences between five tobacco lines and the treatments.

To check the oxidative damage, we investigated the generation of ROS in plants subjected to a hypoxic (near anoxic, ~0.001% O_2_) atmosphere. The level of O_2_^−^ in tobacco leaves was significantly higher in the knockdowns than in other plant lines under normal conditions. The O_2_^−^ level increased significantly in response to a 6 h hypoxia treatment and was the highest under this treatment in the knockdowns and the lowest in the overexpression plants, with the WT showing an intermediate amount (Figure 2A). H_2_O_2_ levels were significantly higher in the overexpressor B8 and the knockdown RI9 than in the other lines under normoxia. After the hypoxic treatment, AOX knockdowns exhibited greater H_2_O_2_ levels than WT, while the overexpression plants had similar H_2_O_2_ levels as the WT (Figure 2B).

In addition to the burst of ROS, the rate of electrolyte leakage (ELR) can also reflect the degree of oxidative damage in tobacco caused by hypoxic condition. AOX overexpressors, particularly B8, displayed less stress damage than other plant lines, according to our findings in Figure 2C. A higher ELR in AOX knockdowns revealed severe oxidative damage.

**Figure 3:**
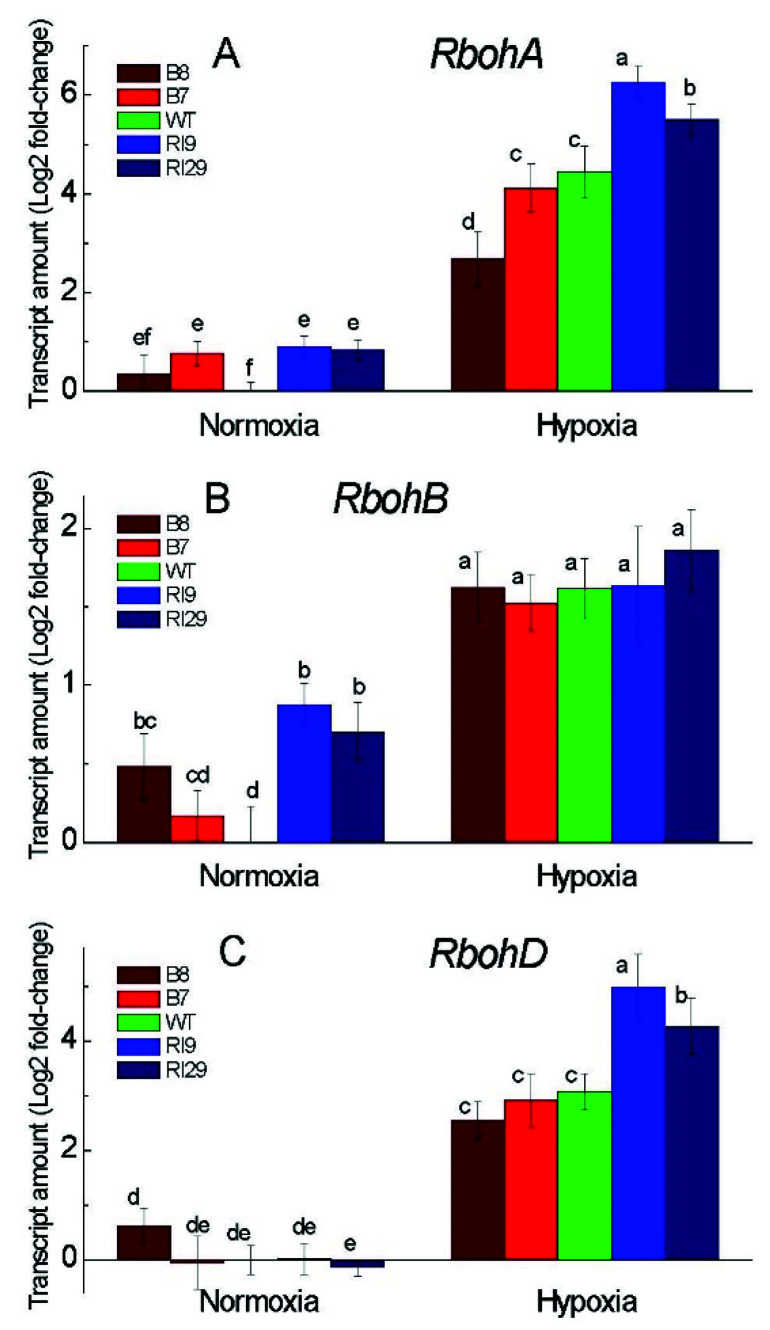
Transcript levels of the genes encoding the subunits RbohA (**A**), RbohB (**B**), and RbohD (**C**) of the respiratory burst oxidase homolog in leaves of tobacco plants exposed to normoxia and 6 h hypoxia. The plant lines used included wild type (WT), two alternative oxidase overexpressors (B7 and B8), and two alternative oxidase knockdowns (RI9 and RI29). Vertical bars indicate SD from three to four independent experiments (*n* = 3–4); different letters indicate significant differences between five tobacco lines and the treatments.

The transcript levels of the NADPH oxidase enzymes (Rbohs) generating ROS, determined by quantitative RT-PCR, revealed the differences between tobacco lines under normoxia and hypoxia. In air, *RbohB* expression was significantly higher in the knockdowns and in the overexpressor B8, as compared to WT (Figure 3B). *RbohA* mRNA levels showed a similar pattern under normoxia, with higher amount in the knockdowns and the overexpressor B7 (Figure 3A). However, the *RbohD* expression was similar across all plant lines relative to the WT (Figure 3C). The transcript levels of these hypoxia-inducible genes were strongly increased after 6 h of hypoxia treatment, and their transcripts were significantly higher in the knockdowns than the WT and the overexpressors. The transcript abundance of *RbohD* and *RbohA* was similar between the WT and overexpression plants under hypoxia, but the level of *RbohA* was somehow lower only in the overexpressor B8 (Figure 3A,C). However, the transcript level of *RbohB* gene was affected under hypoxic stress, with a similar level of increase in all plant lines (Figure 3B).

### 2.3. Antioxidant Enzymes Capacity of Tobacco under Hypoxia

**Figure 4:**
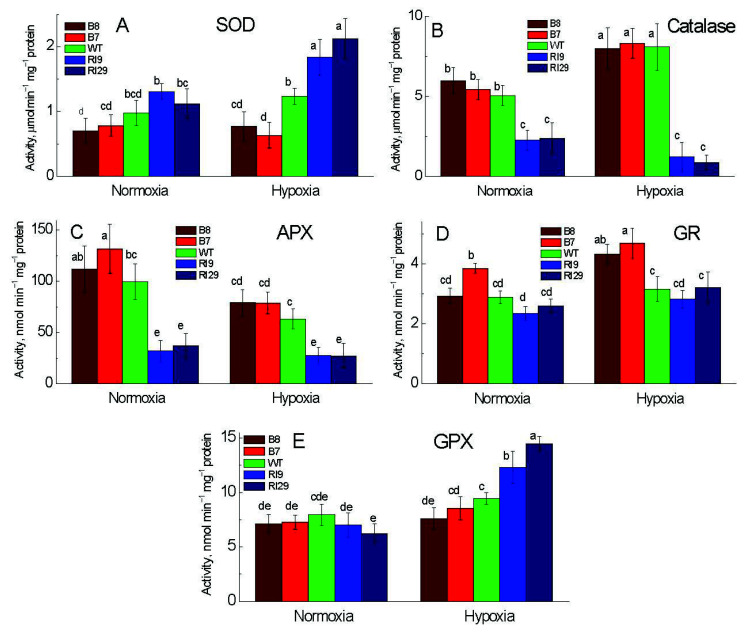
Activities of antioxidant enzymes in leaves of tobacco plants exposed to normoxia and 6 h hypoxia: (**A**) superoxide dismutase (SOD), (**B**) catalase; (**C**) ascorbate peroxidase (APX), (**D**) glutathione reductase (GR), and (**E**) guaiacol peroxidase (GPX). The plant lines used included the wild type (WT), two alternative oxidase overexpressors (B7 and B8), and two alternative oxidase knockdowns (RI9 and RI29). Vertical bars indicate SD from three to four independent experiments (*n* = 3–4); different letters indicate significant differences between five tobacco lines and the treatments.

We monitored the activities of antioxidant enzymes, which play important roles in plants to mitigate oxidative damage. In our study, we investigated the activities of superoxide dismutase (SOD), catalase (CAT), guaiacol peroxidase (GPX), ascorbate peroxidase (APX), and glutathione reductase (GR) (Figure 4). In air, the AOX-upregulating plants and WT exhibited higher activities of CAT and APX as compared to the lower activity in the knockdowns; meanwhile, the SOD activity in the knockdowns was significantly higher than in overexpressors. Under normal air, no significant differences in GPX and GR activity were observed across all plant lines, with the exception of significantly higher GR activity in the overexpressor B7.

While the activity of SOD increased substantially in response to a 6 h hypoxia treatment in the knockdowns, the activity in the overexpressors and WT showed no significant changes (Figure 4A). CAT, as a common antioxidant enzyme present almost in all living tissues, increased significantly under hypoxia in the overexpressors and WT, but no significant changes of CAT activity were observed in the knockdowns (Figure 4B). In response to 6 h hypoxia, the activities of APX and GR were drastically altered. APX activity in the overexpressors reduced significantly but remained greater than in the knockdowns (Figure 4C). Under hypoxia, GR activity increased substantially in the overexpression plants, while the WT and the knockdowns revealed no significant changes and displayed similar activity (Figure 4D). The activity of GPX, as a vital enzyme for the detoxification of H_2_O_2_, followed a similar pattern as SOD, with the greatest activity being in the knockdowns under hypoxia (Figure 4E).

### 2.4. Fermentation under Hypoxia

**Figure 5:**
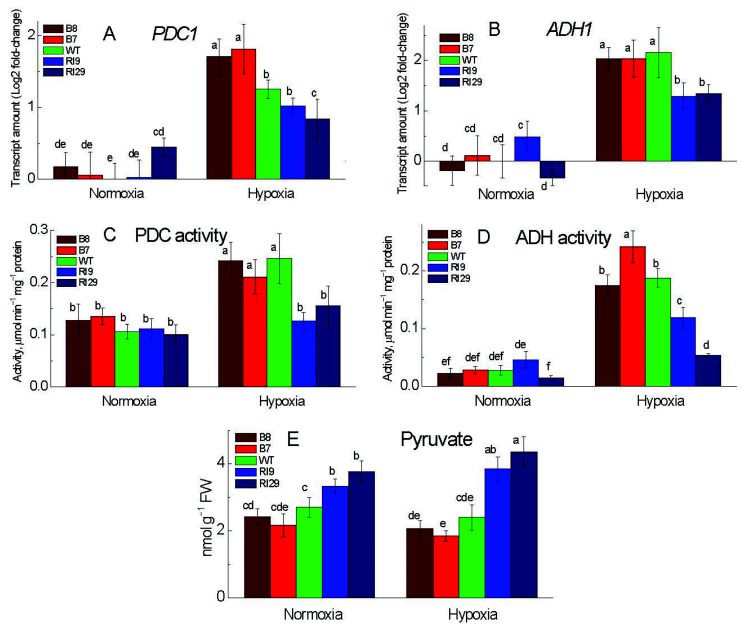
Transcript levels (**A**,**B**) and activities (**C**,**D**) of fermentation enzymes and pyruvate levels (**E**) in leaves of tobacco plants exposed to normoxia and 6 h hypoxia. (**A**,**C**) Pyruvate decarboxylase (PDC) and (**B**,**D**) alcohol dehydrogenase (ADH). The plant lines used included the wild type (WT), two alternative oxidase overexpressors (B7 and B8), and two alternative oxidase knockdowns (RI9 and RI29). Vertical bars indicate SD from three to four independent experiments (*n* = 3–4); different letters indicate significant differences between five tobacco lines and the treatments.

To examine the effect of the AOX transgenes on the ethanol fermentation pathway, the mRNA levels of *PDC1* and *ADH1* (Figure 5A,B) and enzyme activities of PDC and ADH (Figure 5C,D) were analyzed in tobacco leaf under aerobic and hypoxic conditions. Under normal air, the ADH and PDC expression and activity did not differ significantly between the WT and the other plant lines, except for *PDC1* expression, which was marginally higher in the knockdowns RI29 (Figure 5A). In response to the anaerobic condition, the activity and relative expression of the hypoxia-responsive genes *ADH1* and *PDC1* increased substantially. *PDC1* transcript abundance was higher in overexpression plants than in WT (Figure 5A), while PDC activity was increased with a similar pattern (Figure 5C). The knockdowns (particularly RI29) had lower expression and activity of PDC relative to WT. ADH expression was elevated to the same level in the WT and overexpressors, whereas ADH activity peaked in the overexpressor B7 (Figure 5B,D). ADH in the knockdowns, on the other hand, showed lower levels of mRNA and enzyme activity (Figure 5B,D).

The amount of pyruvate was significantly higher in the knockdowns under both aerobic and anaerobic conditions (Figure 5E). Following oxygen deprivation, the level of pyruvate did not differ significantly, except for the knockdown RI29, where it increased as compared to its level in normal air.

### 2.5. Enzymes of Ethylene Synthesis and ERF Induction under Hypoxia

**Figure 6:**
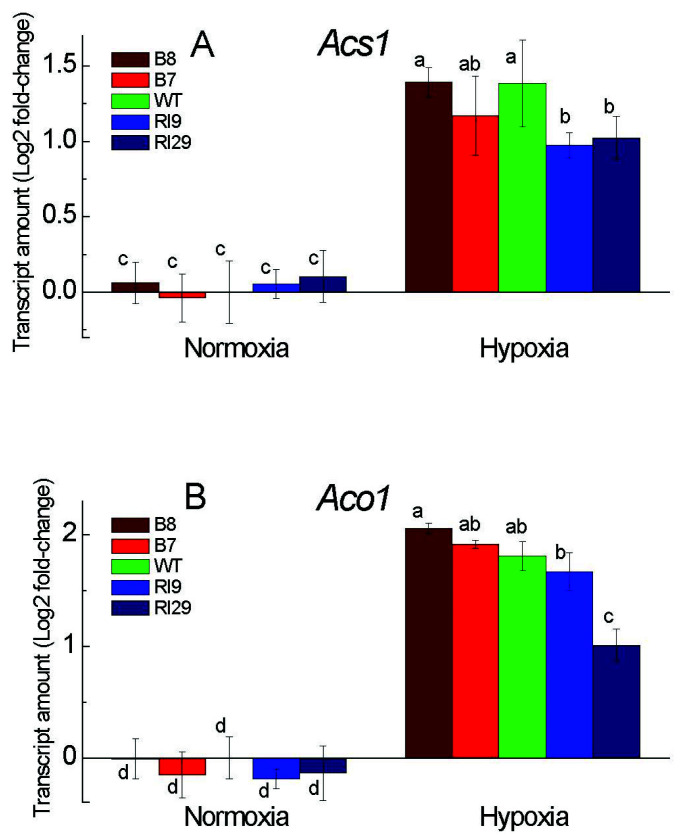
Transcript levels of the genes encoding 1-aminocyclopropane-1-carboxylic acid synthase (*Acs1*) (**A**) and 1-aminocyclopropane-1-carboxylic acid oxidase (*Aco1*) (**B**) in leaves of tobacco plants exposed to normoxia and 6 h hypoxia. The plant lines used included the wild type (WT), two alternative oxidase overexpressors (B7 and B8), and two alternative oxidase knockdowns (RI9 and RI29). Vertical bars indicate SD from three to four independent experiments (*n* = 3–4); different letters indicate significant differences between five tobacco lines and the treatments.

In response to hypoxia, the transcripts abundance of the enzymes of ethylene synthesis 1-aminocyclopropane-1-carboxylic acid synthase (ACS) and aminocyclopropane-1-carboxylic acid oxidase (ACO) strongly increased in the WT and AOX overexpression plants to the similar levels, with the knockdowns (particularly RI29) showing slightly lower increases (Figure 6A,B).

**Figure 7:**
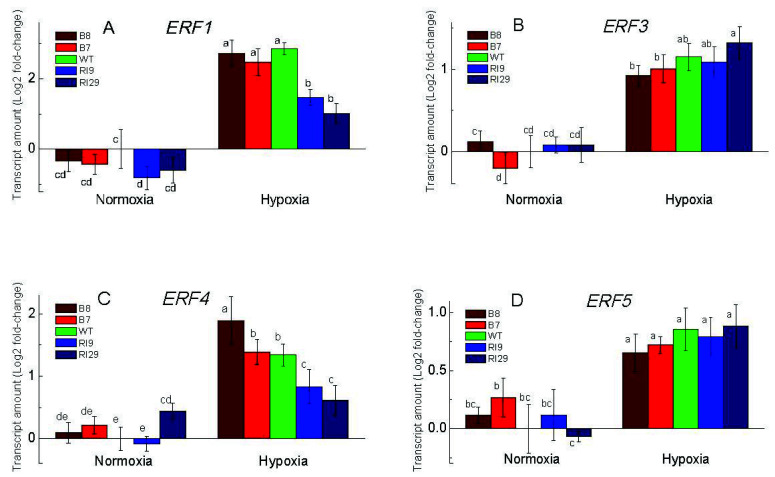
Transcript levels of the genes encoding different ethylene-responsive factors ERF1 (**A**), ERF3 (**B**), ERF4 (**C**), and ERF5 (**D**) in leaves of tobacco plants exposed to normoxia and 6 h of hypoxia. The plant lines used included the wild type (WT), two alternative oxidase overexpressors (B7 and B8), and two alternative oxidase knockdowns (RI9 and RI29). Vertical bars indicate the SD from three to four independent experiments (*n* = 3–4); different letters indicate significant differences between five tobacco lines and the treatments.

The expression of four factors of the ethylene response factor family (ERF1, ERF3, ERF4, and ERF5), which perceive low-oxygen signals and play a crucial role in determining survival in low-oxygen environments (Figure 7), also showed the response to hypoxia, which in some cases was affected by AOX expression. In air, all plant lines exhibited almost the same low expression level of all ERFs tested in this study. The hypoxic treatment increased ERFs transcripts abundance in all plant lines, compared to normoxia. After 6 h of hypoxia, *ERF1* and *ERF4* transcript amounts were significantly higher in the overexpression plants and WT, as compared to the knockdowns, showing the lower transcript amount (Figure 7A,C). All plant lines showed similar high mRNA levels of *ERF3* and *ERF5* under hypoxia treatment (Figure 7B,D).

## 3. Discussion

### 3.1. NO Metabolism under Hypoxia

Nitric oxide (NO) is a crucial signaling molecule in plants since it is quickly produced, stimulates particular activities within cells, and is rapidly scavenged. The AOX knockdown plants have higher levels of leaf NO in normoxia relative to WT tobacco plants. The higher amount of protein *S*-nitrosylation can be linked to an increase in NO generation in this plant line. The presence of AOX can reduce electron transfer across complexes III and IV under normoxia, avoiding electron leakage to nitrite and subsequent NO accumulation in AOX overexpressors; this was directly estimated in planta, using laser scanning fluorescent confocal microscopy and biochemical methods [4].

In contrast to its regular function under normoxia, AOX plays a unique role in hypoxia, where it can increase nitrite-dependent NO production [17,18]. AOX overexpression plants have higher NO production compared to AOX knockdown under hypoxia (Figure 1A,B). Endogenous NO plays an important role in the regulation of target proteins by post-translational modifications (PTMs). NO-mediated *S*-nitrosylation of various proteins, including ERF-VIIs, COX, aconitase, and APX, might be connected to flooding signaling and tolerance [10,18]. Furthermore, NO can activate ethylene production, potentially by the *S*-nitrosylation of important enzymes such ACS and ACO [19]. The *S*-nitrosylation of RbohD reduces its function, limiting the cell death caused by stress-induced oxidative bursts [20]. Given the substantial role of NO in gene regulation, metabolism, and physiology, it is reasonable to assume that AOX supports continued NO production, maybe through the efficient operation of the Pgb-NO cycle. Pgb1 expression was shown to be higher in AOX overexpressors and lower in AOX knockdowns according to our recent study [2]. Under hypoxia, the crosstalk between AOX and NO drives the Pgb-NO cycle, which modulates NO metabolism, influencing the function of target genes/proteins [2].

### 3.2. Oxidative Damage under Hypoxia

The ROS levels change in a dynamic and rapid manner in plant tissues under environmental stress. Under normal air, AOX knockdown increased the leaf amount of O_2_^−^ (Figure 2A), implying that AOX respiration is critical in limiting ROS production by the mitochondrial ETC. Apart from the knockdowns, the overexpressor B8 enhanced the amount of H_2_O_2_ in the leaves under normoxia, revealing an intriguing and convoluted relationship between AOX and H_2_O_2_ levels (Figure 2B). NADPH oxidase, which is encoded by the *Rboh* genes, is a major generator of ROS in plants [3]. Under normal air, we found that *RbohA* and *RbohB* transcript levels were slightly greater in the knockdowns and also in one of the overexpressors; this could explain the high amount of H_2_O_2_ under normoxia.

Following hypoxia, which was measured immediately after the hypoxic treatment, both reactive species rose; and O_2_^−^ and H_2_O_2_ levels were substantially lower in the overexpressors and much higher in the knockdowns, as compared to WT, indicating the important role for AOX in keeping ROS levels under control by regulating the production and removal systems of ROS. After 6 h of hypoxia, we observed the high expression of all Rbohs genes tested in this work (Figure 3). The pattern of the H_2_O_2_ level across plant lines was mirrored by the expression levels of Rbohs (especially *NtRbohD* and *NtRbohA*), which were also elevated in the knockdowns and reduced in the overexpressors in comparison to WT (Figure 2B and Figure 3). In our research, a higher level of AOX decreased the production of H_2_O_2_ in overexpressors under hypoxia through regulating the expression of the *Rboh* genes. Both ROS can act as signaling molecules when produced in a tightly controlled manner, or as cell damaging factors when produced in an uncontrolled fashion [21]. Superoxide anion can interact with NO, initiating the ROS-dependent NO degradation pathway that involves thioredoxins [22]. H_2_O_2_ can activate the expression of ERF73 and ADH, via modulation of ET signaling under oxygen deprivation [23]. By controlling the expression of Rbohs, we assume that AOX determines the signal’s strength and specificity by keeping H_2_O_2_ at a relative level, which has to be elucidated further in this hypoxic model. Electrolyte leakage is widely used as an indicator of membrane damage caused by produced ROS. The ELR was lower in plants expressing AOX, thus clearly indicating that AOX alleviates anaerobic-stress-induced oxidative damage by controlling ROS production.

### 3.3. Antioxidant Defense System under Hypoxia

Antioxidant enzymes scavenge superfluous ROS caused by stress conditions and protect plants from oxidative damage. The increases in antioxidant systems have been reported in the plants lacking AOX under stress circumstances [24,25]. Following hypoxia, the activity levels of SOD and GPX were shown to be significantly greater in the transgenic plants lacking AOX (Figure 4), correlating with higher levels of superoxide anion. Giraud et al. [26] demonstrated that, in the *AOX1a* T-DNA insertion lines, the expression of FeSOD2, FeSOD3, and other antioxidant enzymes was upregulated. These findings revealed that, whereas AOX may directly restrict ROS formation, other antioxidant defense systems can supplement this effect in knockdowns. H_2_O_2_ levels need constant control to prevent hydroxyl radical production via Fenton chemistry, in particular, when CAT levels are low in the conditions of hypoxia/anoxia. In our work, a significant increase in CAT activity was observed in the plants overexpressing AOX. Other investigations observed the increases in the activity of numerous antioxidative enzymes under waterlogging/flooding, such as APX [27], GR, and CAT [28].

Following hypoxia, GR activity was considerably higher in the overexpressors than in the other plant lines. Under hypoxia, the highly reducing circumstances could be mirrored by the increased GR activity, keeping the antioxidants (ascorbate and glutathione) in their physiologically active and reduced states through the oxidation of NADH and NADPH [27]. We presume that the antioxidant defense systems can be induced by ROS generation when the lack of AOX takes place. However, the high basal activities of antioxidant defense systems in the overexpressors under any conditions, aerobic and anaerobic, indicates that AOX maintains the function of the antioxidant enzymes, to some extent, in favor of the antioxidant-to-oxidant ratio.

### 3.4. Fermentation under Hypoxia

In hypoxia, a combination of glycolysis stimulation and the TCA cycle slowing may raise pyruvate levels [29]. Pyruvate accumulation is hypothesized to trigger AOX activity, which stimulates the TCA cycle carbon flow and so reduces pyruvate levels [30]. Our findings back this up by revealing that the plants that upregulate AOX had lower pyruvate levels (Figure 5E), meaning that pyruvate moved into fermentation pathways. The metabolic transition in plants may be caused by the acidification of cytoplasm during anoxia, as well as by pyruvate moving into the fermentation pathway [31]. The upregulation of fermentation pathways for energy production by boosting ADH and PDC activity is one of the earliest and best-studied responses to low oxygen [32,33]. Following hypoxia, *PDC1* and *ADH1* transcripts increased considerably in all plant lines studied here, with highest level in overexpressors (Figure 5). It indicates that AOX improves the activity of PDC and ADH indirectly, presumably through the binding of hypoxically induced ERFs to their gene’s promoters. In the plants overexpressing AOX, this leads to the enhanced ATP and NAD^+^ production.

### 3.5. Ethylene Synthesis Enzymes and ERF Induction under Hypoxia

ET is synthesized from *S*-adenosyl-L-methionine (SAM) via 1-aminocyclopropane 1-carboxylic acid (ACC) in the reactions catalyzed by ACS and ACO. Here, the transcript levels of *ACS1* and *ACO1* were higher in the plants expressing AOX (Figure 6). We believe that AOX-induced NO can boost ET production by inducing ACO and ACS activity via *S*-nitrosylation. In turn, the generated ET can stimulate Pgb production, which regulates NO metabolism, as revealed by van Veen et al. [34] and Hartman et al. [11]. The ubiquitin-mediated protein degradation regulates ERFs protein stability via the 26S proteasome pathway. NO targets ERF-VII for proteasomal degradation through an O_2_-dependent N-end rule pathway [11]. Since NO-mediated PTMs have a strong relationship with ubiquitylation-mediated proteasomal degradation of proteins, we hypothesize that AOX-induced Pgb impacts the ubiquitination and degradation of other groups of ERFs by modulating NO levels.

Deprivation of oxygen increases the activity of ACSs, thus increasing ET synthesis, and it activates downstream genes such as *ERF73* and *ADH1* [35]. ET accumulation leads to *ERFs* (particularly ERF-VII) gene induction, and ERFs participate in *Arabidopsis* defense downstream of ET signaling [36,37]. ERFs, on the other hand, could be regulators of ACO expression, as reported in tomato, banana, and rice [38,39,40]. AOX-induced ET production as a result of hypoxia-induced elevated redox levels leads to ERFs’ activation and is controlled by ERFs feedback mechanisms which in turns control cellular ET level.

In this study, hypoxia increased the number of ERF transcripts in all plant lines. The examined *ERF1* and *ERF4* positively correlated with the overexpression of AOX under hypoxic stress. Studies show that ERFs’ genes (particularly ERF-VII) are key factors in improving plant tolerance to hypoxia by increasing anaerobic gene expression, ADH, PDC, and ROS metabolizing enzymes [8,41,42]. A lack of AOX may lead to leaf PCD in response to hypoxia, as indicated by the severe oxidative damage found in this study, with elevated ROS and ELR in the knockdowns. According to Ogata et al. [43], PCD induction may be mediated by the tobacco transcriptional repressor ERF3, for which the expression is somewhat higher in the AOX knockdown, RI29 (Figure 7). We assume that ERFs, particularly ERF1 and ERF4, as ET signaling pathway markers can either activate or suppress the genes encoding fermentation enzymes, Rbohs, and antioxidant enzymes through directly binding to their promoters.

**Figure 8:**
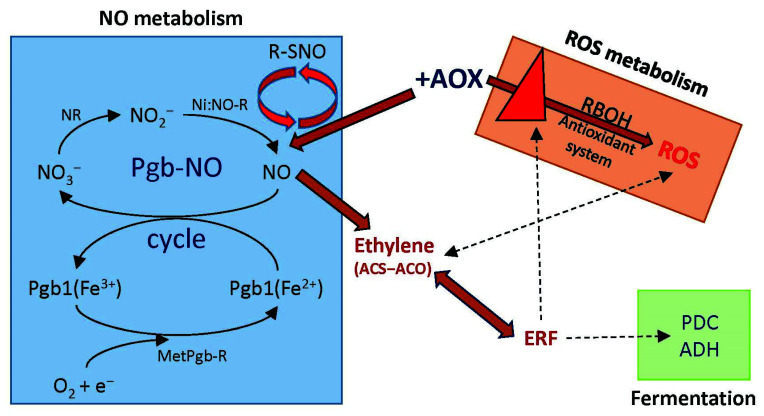
A model for the role of AOX in the activation and regulation of NO, ROS, and ethylene to improve tobacco resistance to hypoxia. Abbreviations: NR, nitrate reductase; Ni:NO-R, nitrite:NO reductase activity of electron transport components; MetPgb-R, reductase of metphytoglobin; PDC, pyruvate decarboxylase; ADH, alcohol dehydrogenase; ERF, ethylene response factors.

From the findings presented in this work, a model can be proposed that explains some of the aspects of stress signaling during anoxia (Figure 8). In this model, AOX levels control the intensity and maybe specificity (H_2_O_2_ vs. O_2_^−^) of the generated ROS signal via modifying the level of Rbohs and antioxidant systems. By damping the electron leak from the ETC and increasing Pgb1, AOX could modulate the intensity of the NO. AOX can induce ET biosynthesis genes and ERFs either directly or indirectly by modulated level of NO and ROS. Produced ET might control the NO production through Pgb1 activation, and induced ERFs can regulate Rbohs and fermentation to keep energy levels stable during anoxia. We propose that AOX regulates the interaction of NO, ROS, and ET in the cell, and these act as signals for the onset of tobacco responses in the absence of oxygen.

## 4. Materials and Methods

### 4.1. Plant Material, Growth Condition, and Hypoxia Treatment 

Tobacco (*Nicotiana tabacum* L. cv. Petit Havana SR1) wild type (WT) and transgenic lines with the suppressed levels of AOX protein (RI9 and RI29) and elevated levels of AOX protein (B7 and B8) were used for all experiments. The transgenic lines used in this work have been described previously [44,45]. RI29 has no detectable leaf AOX protein, while very low amounts of leaf AOX protein can still be detected in RI9, and the overexpressor B8 shows slightly higher amounts of AOX protein than B7 [44,45]. The plants were grown in a cultivation chamber under controlled environmental conditions for 4 weeks before tests, as previously described [46]. To gain a full understanding of how AOX impacts the ROS metabolism under oxygen deficiency, plants were exposed to a nitrogen atmosphere, and the samples were taken at 0 h (normoxia) and 6 h of the hypoxic condition. To test the plants under low oxygen, an entire individual plant was placed in a custom-built sealed dark chamber, where the air supply could be replaced with nitrogen gas, having ~0.001% oxygen [47]. By depriving plants of oxygen and light, the treatment eliminates both aerobic respiration and photosynthesis. After hypoxia, the tobacco leaves (especially of the downregulating lines) often displayed irregular margins, bent petioles associated with smaller-than-normal downward wilted leaves, as shown in the previous study [46]. The control plants were treated with normal air in the same chamber. The fourth leaf from the top of each plant was taken after treatment, instantly frozen, and kept in liquid nitrogen before being transferred to the −80 °C freezer. The images of AOX overexpression (B7 and B8), WT, and knockdowns (RI9 and RI29) under normoxia and after anoxic treatment were presented in the previous study [46].

### 4.2. Reactive Nitrogen and Oxygen Species

NO was measured in the gas phase, using the chemiluminescence detection method, as described before [48]. The excised leaves were placed in 20 mM Hepes buffer (pH 7.0) containing 50 mM sodium nitrate before being transferred to a glass chamber with a continuous steady input of nitrogen at 120 mL min^−1^. A vacuum pump coupled to an ozone destroyer drew the measuring gas (purified air or nitrogen) through the chemiluminescence detector (CLD 88 p; Eco-Physics, Dürnten, Switzerland). The measuring gas was made NO free by a NO scrubber (Eco Physics Ltd., Dürnten, Switzerland). All gas flows were adjusted by using flow controllers (Thermo Fisher Scientific, Waltham, MA, USA).

NO in the liquid phase was measured by the oxyhemoglobin assay. NO was extracted from frozen leaves of tobacco with 1 mL of cooled buffer (50 mM Tris-HCl, pH 7.0, 0.6% (*w*/*v*) PVP). The homogenates were centrifuged at 15,000× *g* for 10 min at 4 °C. The supernatant was pretreated for 3 min, at room temperature, with superoxide dismutase (SOD; 4000 U/mL) and catalase (10,000 U/mL) to remove ROS. NO was quantified in cleared extracts by spectrophotometrically measuring the conversion of oxyhemoglobin to methemoglobin [49].

To measure the amount of superoxide anion (O_2_^−^), 200 mg of fresh leaf biomass was crushed by using a pestle and mortar in 2 mL of 8 M KOH under chilled conditions, and then it centrifuged for 15 min at 12,000× *g* at 4 °C. The amount of O_2_^−^ in the supernatant was then evaluated at 550 nm by reduction of cytochrome *c*, as described in Ma et al. [49]. The H_2_O_2_ content of leaves was measured as described by Xu et al. [50]. Approximately 200 mg fresh leaf biomass was homogenized in an ice bath with 5 mL 0.1% TCA and centrifuged for 20 min at 12,000× *g* and 4 °C. Then 0.5 mL of supernatant was added to 0.5 mL of 10 mM potassium phosphate buffer (pH 7.0) and 1 mL 1M KI, and the absorbance was read at 390 nm.

### 4.3. Assays for Antioxidant Enzymes

Fresh leaf tissue (300 mg) was homogenized with 3 mL of ice-cold 25 mM HEPES buffer (pH 7.8) containing 0.2 mM EDTA, 2 mM ascorbate, and 2% PVP. The homogenates were then centrifuged for 20 min at 12,000× *g* at 4 °C. The resulting supernatants were used to determine the enzymatic activities of SOD, CAT, GPX, APX, and GR. SOD (EC 1.15.1.1) activity was assayed by measuring the inhibition of the photochemical reduction of NBT, following the method of Wu et al. [51]. CAT (EC 1.11.1.6) activity was determined as the decline in the absorbance at 240 nm due to the decrease of extinction of H_2_O_2_, using the method of Huang et al. [52]. GPX (EC 1.11.1.7) activity was measured as the increase in the absorbance at 470 nm due to guaiacol oxidation [53]. APX (EC 1.11.1.11) activity was measured by tracking the drop in absorbance at 290 nm as ascorbate was oxidized [54]. GR (EC 1.8.1.7) activity was monitored at 340 nm for 3 min by the rate of conversion of NADPH to NADP^+^ (ε_340_ = 6.2 mM^−1^ cm^−1^) [49].

### 4.4. Assays of Fermentation Enzymes and Metabolites

Pyruvate decarboxylase (PDC; EC 4.1.1.17) activity was evaluated by measuring NADH oxidation in a coupled reaction with alcohol dehydrogenase (ADH) [55]. The reaction mixture consisted of 50 mM MES-KOH buffer, pH 6.5, 5 mM MgCl_2_, 0.5 mM TPP, 6 mM pyruvate, 0.67 mM NADH, 1 mM DTT, and 3.5 units mL^−1^ of yeast ADH (Sigma-Aldrich, St. Louis, MO, USA). The reaction was initiated by adding protein extracts, and the absorbance was read at 340 nm.

Alcohol dehydrogenase (ADH; EC 1.1.1.1) activity was measured by monitoring at 340 nm the reduction of NAD^+^ during ethanol oxidation, using an assay buffer of 0.5 M Tris-HCl, pH 9.0, 0.1 M ethanol, and 2 mM NAD^+^ [56].

To measure the amount of pyruvate, freshly frozen biomass (100 mg) was gently homogenized in 1 mL 70% HClO_4_. The homogenate was then neutralized by using 5 M KOH and then centrifuged at 7000× *g* for 10 min at 4 °C. The clear supernatant was used for spectrophotometric determination of pyruvate by the enzymatic assay coupled to NADH oxidation, as described in Dinakar et al. [57].

### 4.5. Protein S-Nitrosylation

Leaf biomass (100 mg) was homogenized in 1.8 mL of 50 mM HEPES-KOH buffer (pH 8.0) containing 0.2% (*w*/*v*) SDS, 0.5% (*w*/*v*) CHAPS, 1 mM EDTA, and 0.1 mM neocuproine (which inhibits denitrosylation). The homogenate was centrifuged (15,000× *g*, 10 min, 4 °C), and protein *S*-nitrosylation was determined by reducing R-SNO to R-SH in the presence of ascorbate and then measuring free thiol groups, using 5,5′-dithio-bis (2-nitrobenzoic acid) [49].

### 4.6. Electrolyte Leakage Rate

The relative electrolyte leakage rate was measured as described by Guo et al. [58]. The leaf discs were placed in 10 mL of distilled water and incubated for 2 h. After measuring the initial electrical conductivity (EC1) of the leaves, the samples were boiled for 30 min to achieve the final electrical conductivity (EC2). The electrolyte leakage rate (ELR) was calculated by using the formula ELR = (EC1/EC2) × 100.

### 4.7. Transcript Amounts 

Total RNA was extracted from frozen leaf, using the FastRNA^®^ Pro Green Kit (MP Biomedicals, Irvine, CA, USA). The first strand of cDNA was synthesized from 5 μg total RNA, using the Superscript III reverse transcriptase kit (Invitrogen). Quantitative PCR was carried out with an Applied Biosystems StepOnePlus Real-Time PCR 128 System, using SYBR Green qPCR Master Mixes (Thermo Fisher Scientific, Waltham, MA, USA) and gene-specific primers (Supplementary Table S1). Triplicate reactions were performed with three biological replicates, and the relative RNA expression was analyzed by using the 2^−ΔCt^ method. The relative transcript abundance of target genes was normalized against the geometric mean of the CT value of two reference genes, *NtACT* and glyceraldehyde phosphate dehydrogenase (GAPDH). Transcripts levels of *NtADH1* (alcohol dehydrogenase 1), *PDC1* (pyruvate decarboxylase), *NtACO1* (1-aminocyclopropane-1-carboxylic acid oxidase), NtACS1 (1-aminocyclopropane-1-carboxylic acid synthase), *NtRbohA,B,D* (plasma membrane NADPH oxidase, or respiratory burst oxidase), and *NtERF1,3,4,5* (ethylene-responsive factors belong to groups IX and VIII (only *ERF3*)) were analyzed in this study.

### 4.8. Statistical Analysis

The software package SPSS V. 21.0 (Statistical Package for Social Science; Chicago, IL, USA) was used for statistical analysis. One-way ANOVA with Duncan’s multiple range was used to identify significant differences between different lines of tobacco. The data in the text and on figures are the means of three biological repeats ± SD. The statistically significant differences at *p* < 0.05 were discussed. 

## 5. Conclusions

The data presented in this study show that AOX expression controls the interaction of NO, reactive oxygen species, and ethylene, triggering a coordinated downstream defensive response against hypoxia.

## Data Availability

The raw data supporting the conclusions of this article will be made available by the corresponding authors, without undue reservation.

## Supplementary Materials

The following is available online at https://www.mdpi.com/article/10.3390/ijms23137153/s1.
