# Supplementary material for: The Role of Alternative Oxidase in the Interplay between Nitric Oxide, Reactive Oxygen Species, and Ethylene in Tobacco (Nicotiana tabacum L.) Plants Incubated under Normoxic and Hypoxic Conditions
Source: Int J Mol Sci. 2022 Jun 28;23(13):7153. doi: 10.3390/ijms23137153 (PMC9266549; doi:10.3390/ijms23137153)
Supplement: Supplementary file 1 [file ijms-23-07153-s001.zip › ijms-1796626-supplementary.pdf]

**Supplementary Table S1.** Primers used for qRT-PCR assays of tobacco leaves

| Primer | Forward sequence | Reverse sequence | Accession no/References |
|---|---|---|---|
| NtACT | TTGGCTTACATTGCTCTTG | TCATTGATGGTTGGAACAG | XM_033660572.1 |
| GAPDH | GGCAGCATCCTTCAACATTATTC | CTCGGAAAGCCATTCCAGTTA | KR007670.1 |
| NtADH1 | AAGCTGGAGGAATTGTGGAGAG | ACCAGTGAACACAGGAAGAACA | Tan *et al.* (2019) |
| NtPDC1 | CCCGAACACCATTCACATTTC | CATCAGCAGATTCCACGATTTC | Tan *et al.* (2019) |
| NtASC1 | AAACGAGCCATTGCAACAAAGA | TGAATCCTGGTAAGCCCATGTC | Tan *et al.* (2019) |
| NtACO1 | GATTACACAAACAGACGGGACT | TTGATTCCACCACACACAATAC | Zhou *et al.* (2008) |
| NtRbohA | AAGGTGTTATGAACGAAGTG | CTGGTGCCTGATACGATA | Xu *et al.* (2018) |
| NtRbohB | CTATGCTTCAGTCTCTTCAC | GGCGTGTTGTCTTAGTTC | Xu *et al.* (2018) |
| NtRbohD | ACCAGCACTGACCAAAGAA | TAGCATCACAACCACAACTA | Wu *et al.* (2008) |
| NtERF3 | AGGAATTGATCTTGATCTTAAC | ACAAAATTCAACCATTAGTCTC | Ogata *et al.*(2012) |
| NtERF5 | GCCCAAAGTTCCAATCTTGA | AACCTCCTGCTGCTTCATT | XM_009596177.3 |
| NtERF4 | CGGGTCTGGTTAGGTACTTTC | GTGACTTGGGCTCTGATTCT | XM_009611511.3 |
| NtERF1 | GGAAGGCATTACAGAGGTGTTA | CCAAACCCTAGCTCCATTCTT | XM_009613506.3 |

**References**


Ogata, T.; Kida, Y.; Arai, T. *et al.* Overexpression of tobacco ethylene response factor *NtERF3* gene and its homologues from tobacco and rice induces hypersensitive response-like cell death in tobacco. *J. Gen. Plant Pathol.* **2012,** ***78,*** 8–17. doi: 10.1007/s10327-011-0355-5

Tan, X.; Zwiazek, J.J. Stable expression of aquaporins and hypoxia-responsive genes in adventitious roots are linked to maintaining hydraulic conductance in tobacco (*Nicotiana tabacum*) exposed to root hypoxia. *PLoS One* **2019,** *14(2),* e0212059. doi: 10.1371/journal.pone.0212059

Wu, L.; Zhang, Z.; Zhang, H.; Wang, X.C.; Huang, R. transcriptional modulation of ethylene response factor protein JERF3 in the oxidative stress response enhances tolerance of tobacco seedlings to salt, drought, and freezing. *Plant Physiol.* **2008,** *148(4),* 1953-1963. doi: 10.1104/pp.108.126813

Xu, Y.; Zheng, X.; Song, Y. *et al.* NtLTP4, a lipid transfer protein that enhances salt and drought stresses tolerance in *Nicotiana tabacum*. *Sci. Rep.* **2018,** *8,* 8873. doi: 10.1038/s41598-018-27274-8

Zhou, J.; Zhang, H.; Yang, Y.; Zhang, Z.; Zhang, H.; Hu, X.; Chen, J.; Wang, X.C.; Huang, R. Abscisic acid regulates TSRF1-mediated resistance to *Ralstonia solanacearum* by modifying the expression of GCC box-containing genes in tobacco. *J. Exp. Bot.* **2008,** *59(3)*, 645-652. doi: 10.1093/jxb/erm353
